# Collaborative model for diagnosis and treatment of very rare diseases: experience in Spain with thymidine kinase 2 deficiency

**DOI:** 10.1186/s13023-021-02030-w

**Published:** 2021-10-02

**Authors:** Cristina Domínguez-González, Marcos Madruga-Garrido, Michio Hirano, Itxaso Martí, Miguel A. Martín, Francina Munell, Andrés Nascimento, Montse Olivé, Joanne Quan, M. Dolores Sardina, Ramon Martí, Carmen Paradas

**Affiliations:** 1 Neuromuscular Disorders Unit, Neurology Department, Hospital 12 de Octubre, Madrid, Spain; 2 Instituto de Investigación imas12, Hospital 12 de Octubre, Madrid, Spain; 3 Center for Biomedical Network Research On Rare Diseases (CIBERER), Instituto de Salud Carlos III, Madrid, Spain; 4 Pediatric Neurology Department, Hospital U. Virgen del Rocío, Seville, Spain; 5 Neurology Department, H. Houston Merritt Center, Columbia University Irving Medical Center, New York, NY USA; 6 Pediatric Department, Donostia University Hospital, Biodonostia Health Research Institute, University of the Basque Country, San Sebastián, Spain; 7 Mitochondrial Diseases Laboratory, Department of Biochemistry, Research Institute Hospital 12 de Octubre (imas12), Madrid, Spain; 8 Pediatric Department, Vall d’Hebron Hospital, Barcelona, Spain; 9 Neuromuscular Unit, Neurology Department, Sant Joan de Déu Research Institute, Sant Joan de Déu Hospital, Barcelona, Spain; 10 Neuromuscular Disorders Unit, Department of Neurology, Hospital de la Santa Creu i Sant Pau/Center for Biomedical Network Research On Rare Diseases (CIBERER), Barcelona, Spain; 11 Zogenix, Inc., Emeryville, CA USA; 12 Pediatric Neurology Department, Badajoz Hospital Complex, Badajoz, Spain; 13 Research Group On Neuromuscular and Mitochondrial Diseases, Vall d’Hebron Research Institute, Autonomous University of Barcelona, Barcelona, Spain; 14 Neurology Department, Neuromuscular Disorders Unit, Instituto de Biomedicina de Sevilla, Hospital U. Virgen del Rocío, CSIC, Universidad de Sevilla, Avd. Manuel Siurot s/n, 41013 Sevilla, Spain; 15 Center for Biomedical Network Research On Neurodegenerative Disorders (CIBERNED), Instituto de Salud Carlos III, Madrid, Spain

**Keywords:** Mitochondrial disease, Mitochondrial medicine, Thymidine kinase 2 deficiency (TK2d)

## Abstract

**Background:**

Mitochondrial diseases are difficult to diagnose and treat. Recent advances in genetic diagnostics and more effective treatment options can improve patient diagnosis and prognosis, but patients with mitochondrial disease typically experience delays in diagnosis and treatment. Here, we describe a unique collaborative practice model among physicians and scientists in Spain focused on identifying TK2 deficiency (TK2d), an ultra-rare mitochondrial DNA depletion and deletions syndrome.

**Main Body:**

This collaboration spans research and clinical care, including laboratory scientists, adult and pediatric neuromuscular clinicians, geneticists, and pathologists, and has resulted in diagnosis and consolidation of care for patients with TK2d. The incidence of TK2d is not known; however, the first clinical cases of TK2d were reported in 2001, and only ~ 107 unique cases had been reported as of 2018. This unique collaboration in Spain has led to the diagnosis of more than 30 patients with genetically confirmed TK2d across different regions of the country. Research affiliate centers have led investigative treatment with nucleosides based on understanding of TK2d clinical manifestations and disease mechanisms, which resulted in successful treatment of a TK2d mouse model with nucleotide therapy in 2010. Only 1 year later, this collaboration enabled rapid adoption of treatment with pyrimidine nucleotides (and later, nucleosides) under compassionate use. Success in TK2d diagnosis and treatment in Spain is attributable to two important factors: Spain’s fully public national healthcare system, and the designation in 2015 of major National Reference Centers for Neuromuscular Disorders (CSURs). CSUR networking and dissemination facilitated development of a collaborative care network for TK2d disease, wherein participants share information and protocols to request approval from the Ministry of Health to initiate nucleoside therapy. Data have recently been collected in a retrospective study conducted under a Good Clinical Practice–compliant protocol to support development of a new therapeutic approach for TK2d, a progressive disease with no approved therapies.

**Conclusions:**

The Spanish experience in diagnosis and treatment of TK2d is a model for the diagnosis and development of new treatments for very rare diseases within an existing healthcare system.

## Background

Patients with rare mitochondrial disorders predominantly manifesting with muscle symptoms experience long diagnostic odysseys [1–4]. Most patients with mitochondrial myopathies will see an average of eight physicians over 8–10 years before receiving a diagnosis [4]. For patients with rapidly progressing, severe disease, this delay between onset of symptoms and initiation of treatment could be fatal or could result in significant morbidity [2].

Spain has been uniquely successful in identifying and treating patients with thymidine kinase 2 deficiency (TK2d), an ultra-rare, autosomal recessive mitochondrial DNA (mtDNA) depletion and deletions syndrome (MDDS). A collaborative partnership, mainly between research centers at the Vall d’Hebron Research Institute (VHIR) in Barcelona (Spain) and Columbia University Irving Medical Center (CUIMC) in New York City (USA), has facilitated understanding of disease pathology and the development of oral nucleosides as potential therapy [5]. Spain continues to lead in identification, diagnosis, and treatment of patients with TK2d due to collaboration and networking through the Spanish National Reference Centers for Neuromuscular Disorders (CSURs). Here, we describe our experience in TK2d to exemplify how CSURs can shorten the diagnostic odyssey and consolidate patient care for a rare mitochondrial disease that requires coordination of highly specialized, multidisciplinary care teams to improve patient outcomes and quality of life.

The objective of this work is to illustrate how CSURs in Spain (1) coordinate research, diagnosis, and treatment efforts within the framework of the Spanish National Health System (SNS), (2) optimize access to care for patients with TK2d, and (3) could serve as a model for diagnosis and treatment of patients with rare neuromuscular diseases throughout the EU.

## Main body

### Treatment of TK2d and rare neuromuscular disease in Spain: the importance of reference centers

In 2015, the Spanish Ministry of Health designated a network of National Reference Centers (CSURs) for Neuromuscular Disorders by *Real Decreto* 1302/2006. The TK2d experience in Spain is a uniquely successful example of a rare disease that has been diagnosed and treated within the CSUR framework [6]. Diagnosis and care for patients with rare diseases are most efficiently organized in reference centers, which can provide specialized assistance, ensure equity of access to care [1], and contribute to gathering and dissemination of knowledge [2]. In Spain, CSURs-NMD have simplified and streamlined the referral and diagnostic process. Referrals of patients with TK2d and other mitochondrial myopathies among national Units of Neuromuscular Disorders within the SNS facilitate rapid identification, diagnosis, and treatment of patients.

### TK2 deficiency disease: clinical features

TK2d is characterized by progressive muscle weakness, with predominant distribution in the proximal, bulbar, and facial muscles together with severe respiratory involvement [7–10]. Age of onset and rate of progression are quite variable, but in all its clinical forms, TK2d clinical manifestations can include proximal muscle weakness, dysphagia, and respiratory insufficiency, leading to premature death in a majority of patients [7, 8, 11]. As is the case for most rare diseases, TK2d is probably underdiagnosed. Approximately 107 molecularly confirmed cases were reported worldwide in the published literature as of 2018 [11], but TK2d incidence is probably underestimated due to early mortality and misdiagnoses [3]. Given the progressive nature of the disease and the availability of effective therapy with oral nucleosides, earlier identification, diagnosis, and treatment are critical to patient prognosis, survival, and functionality in the motor, respiratory, and feeding domains [7, 8, 10, 12, 13].

### Collaborative care model in Spain: focus on TK2d

**Fig. 1:**
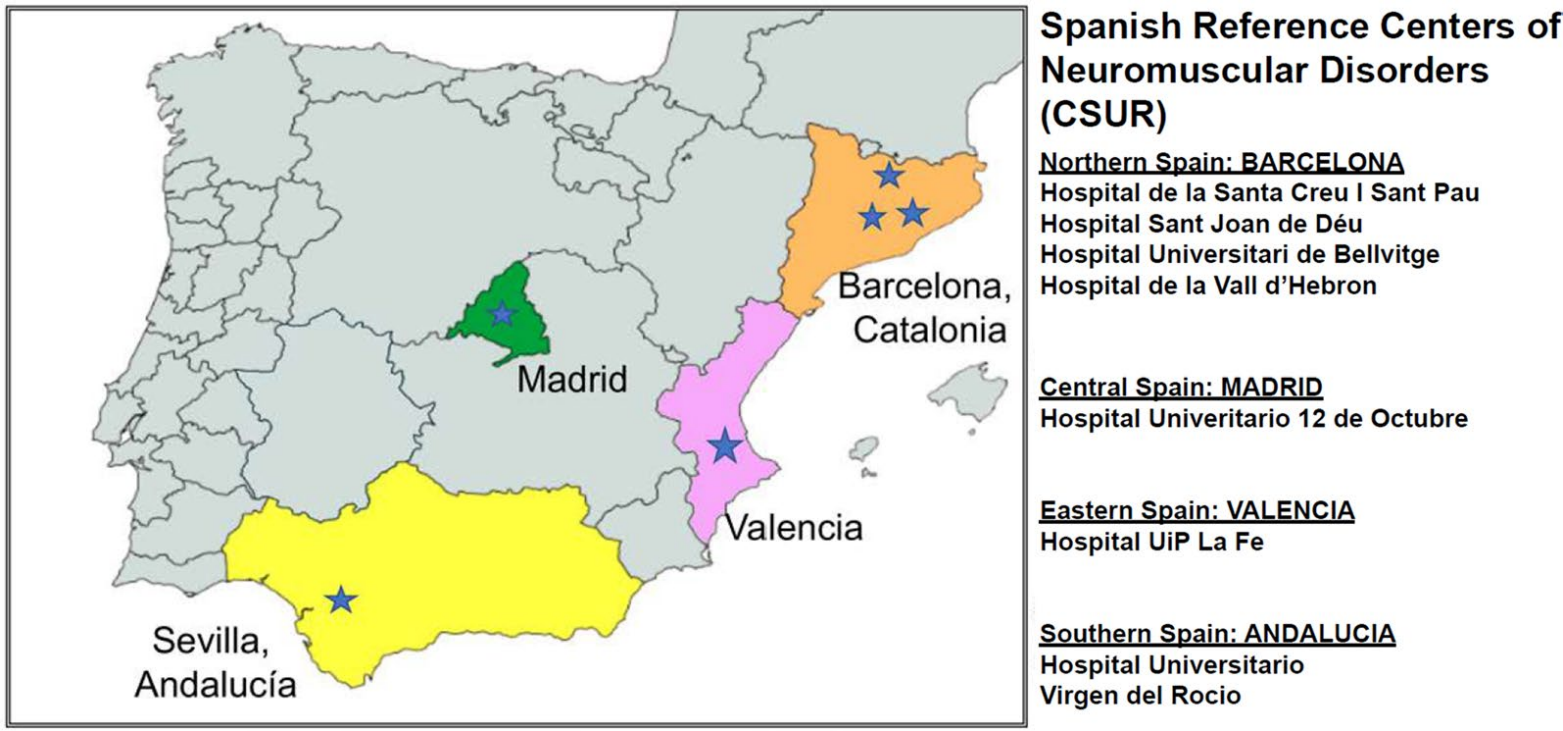
Collaboration among major reference centers across regions of Spain and research affiliates

**Fig. 2:**
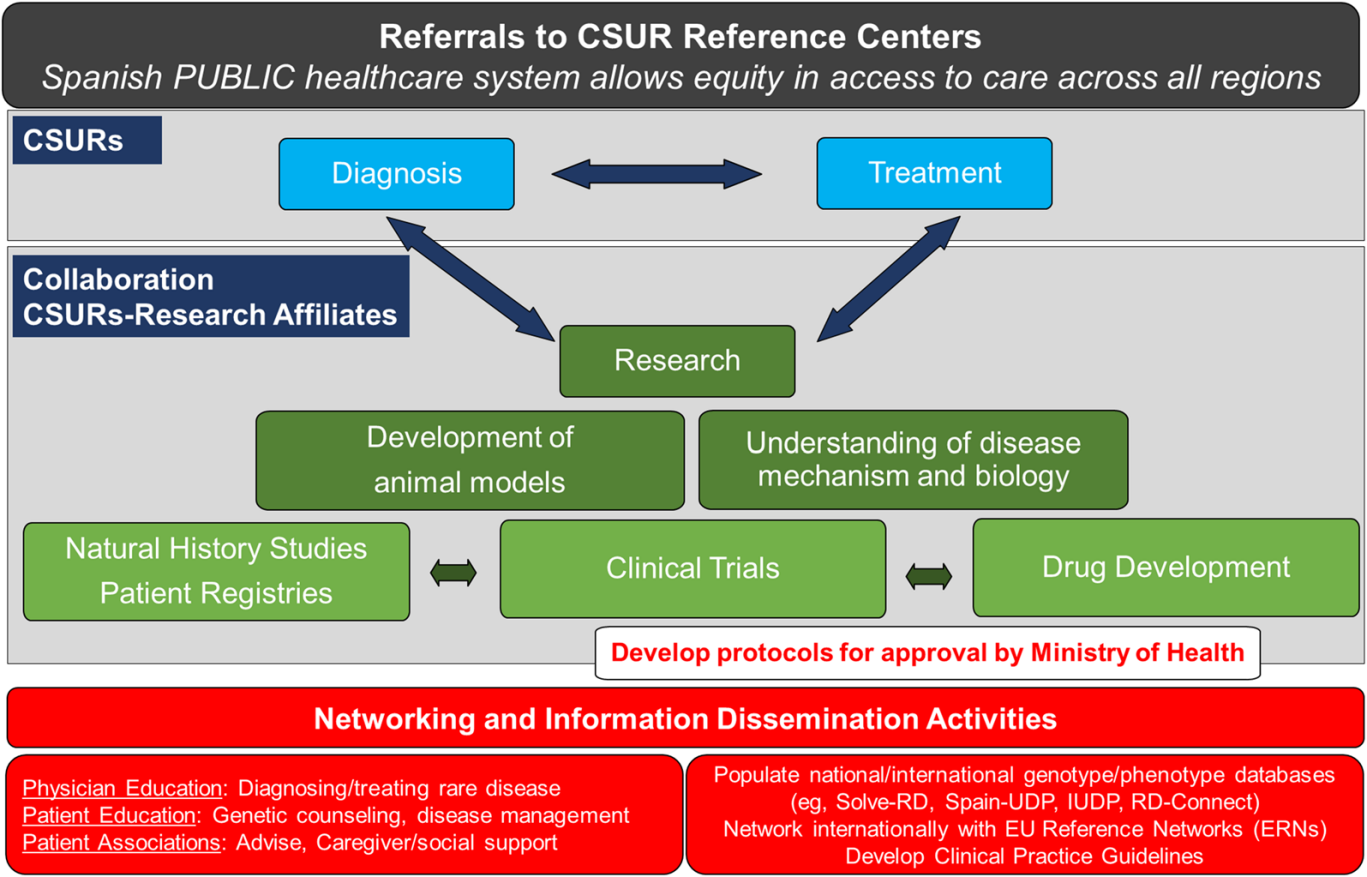
Role of the CSURs in coordinating research, diagnosis, clinical care, and information dissemination

The collaborative care model in Spain has identified and treated the largest cohort of patients worldwide: ~ 30 of the ~ 107 total TK2d cases were diagnosed and treated in Spain (~ 30%). In Spain, most patients with genetic myopathies are referred to CSURs for neuromuscular disorders (CSURs-NMD) for diagnosis and treatment through national neuromuscular units (Fig. 1) [1, 6]. The network of CSURs-NMD throughout Spain coordinate with the Spanish collaboration among national Units of Neuromuscular Disorders to facilitate identification and homogeneous treatment (Fig. 2). CSURs-NMD at Hospital U 12 Octubre (H12O) in Madrid and Hospital U Virgen del Rocío (HUVR) in Sevilla were pioneers in coordinating the process to harmonize research, clinical care, and management of TK2d cases.

Diagnosis and treatment paradigms within the CSUR framework integrate ongoing research and development activities into clinical practice. Collaboration occurs between local centers and CSURs within a regional network, which connects to the larger, national CSUR network. CSURs-NMD further coordinate with research affiliates in Spain (Center for Biomedical Network Research on Rare Diseases [CIBERER], Center for Biomedical Network Research on Neurodegenerative Disorders [CIBERNED], Institute of Biomedicine in Seville [IBiS], Research Institute Hospital 12 de Octubre [imas12], VHIR). Neuromuscular disease specialists provide expert opinion on obtaining differential diagnosis of TK2d, including recommendations for additional testing. CSURs develop clinical protocols and provide treatment for TK2d through clinical trials and compassionate use programs to ensure timely access to care. CSURs-NMD, as part of the wider EURO-NMD, also disseminate information and house registries for international access. Although no archival registry of genetic mutations in TK2d is available, patients with TK2d and their caregivers are invited to join the TK2d patient registry (https://www.tk2d.com/) to facilitate networking and data dissemination. CSURs consolidate expertise to provide care of the highest standard for affected individuals. In the case of TK2d, the Spanish collaboration and CSURs-NMD allowed rapid transition of targeted nucleoside therapy through the development pipeline by coordinating preclinical and clinical research activities, including the recent Good Clinical Practice (GCP)-compliant retrospective protocol. Ongoing research initiatives seek to optimize patient treatment strategies (NCT03845712; NCT03701568). Notably, of 38 total patients enrolled, 23 patients were from Spain (60%). Four of the eight (50%) participating sites were from the collaboration, and patients are continuing to receive nucleoside therapy.

### Key milestones in TK2d diagnosis and treatment: role of CSURs-NMD and Spanish research affiliates

**Fig. 3:**
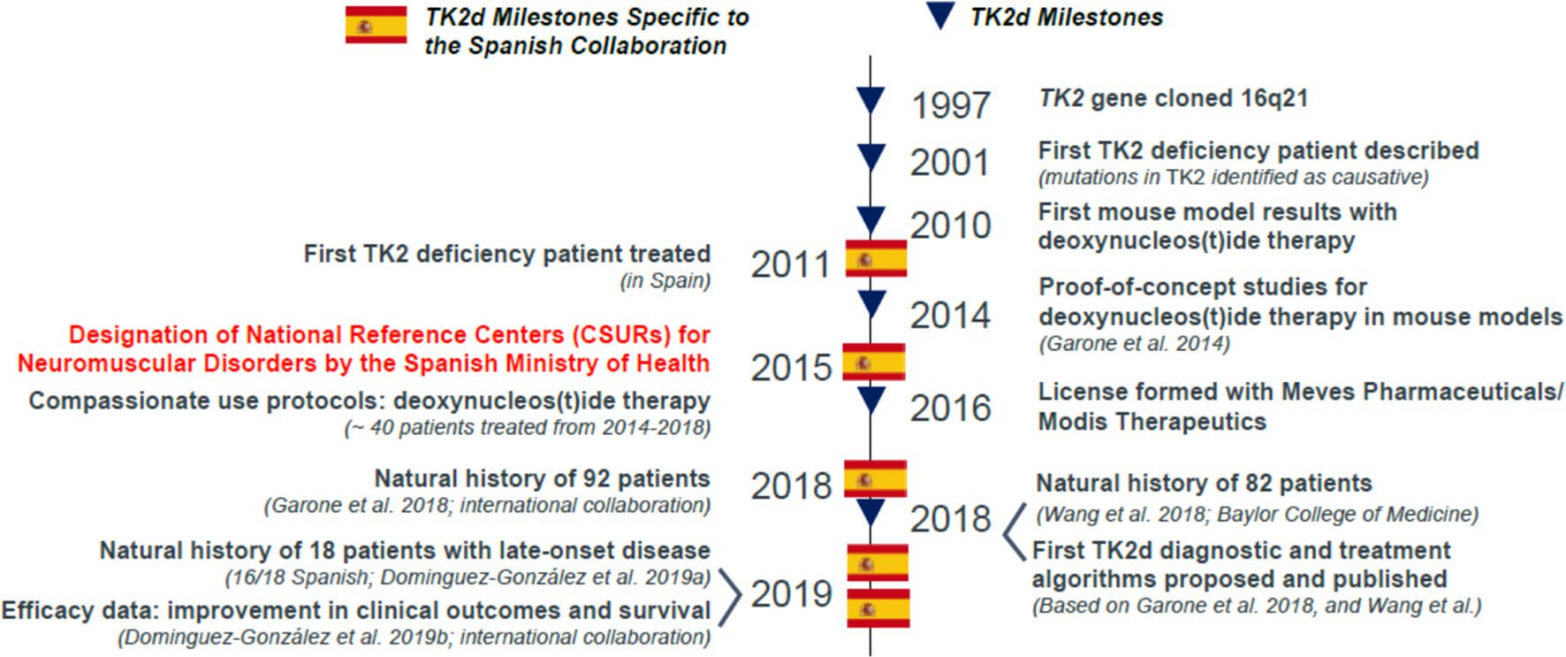
Key milestones in TK2d diagnostics and treatment: role of the Spanish research affiliates and collaborators [7, 10, 14]

**Fig. 4:**
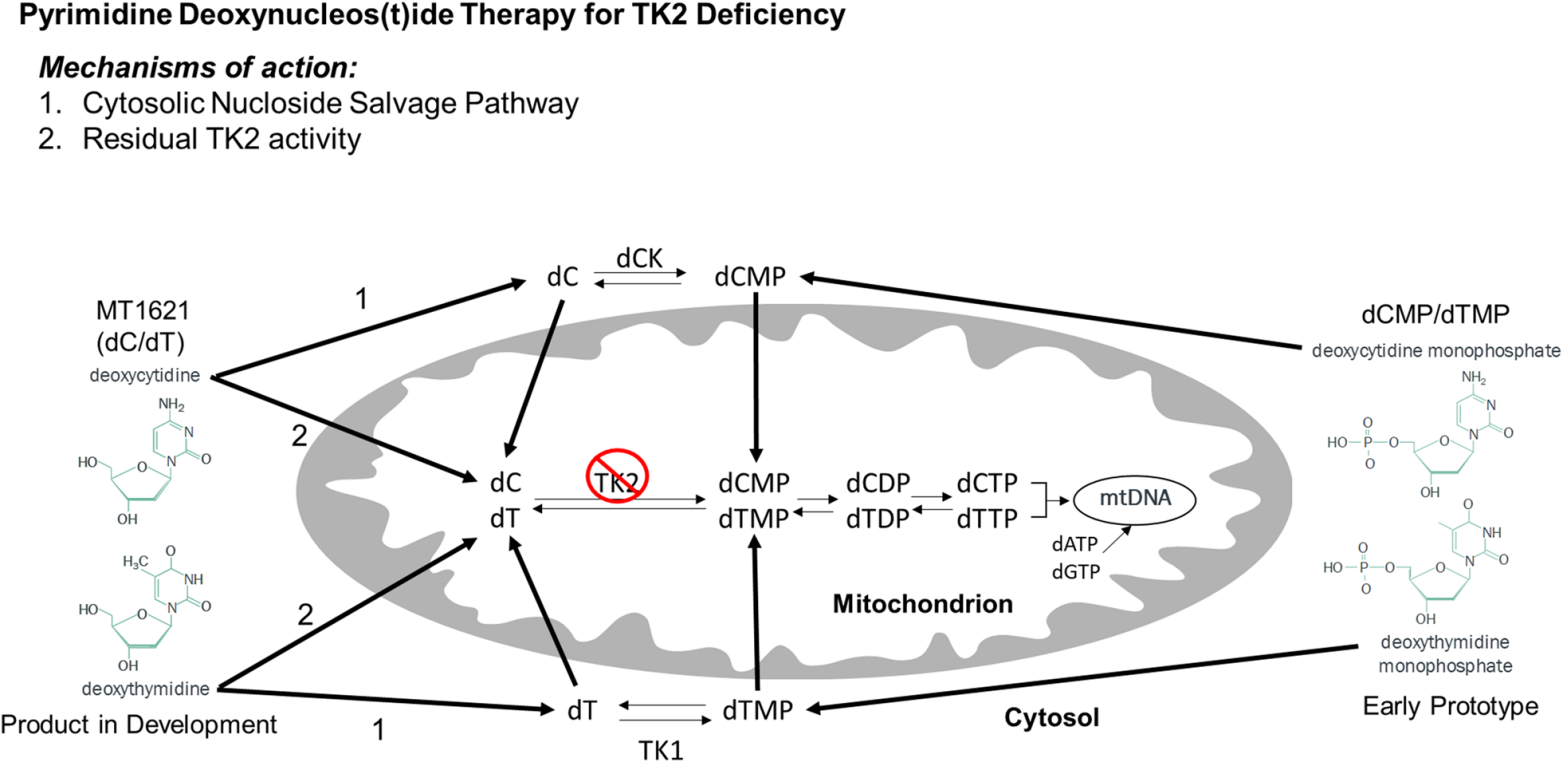
Pharmacological mechanism of nucleoside therapy in TK2d. MT1621 (dC/dT) deoxynucleosides drive production of dTMP and dCMP from the enzymes thymidine kinase 1 (TK1) and deoxycytidine kinase (dCK), respectively, which cross the mitochondrial membrane to provide substrate for dTTP and deoxycytidine triphosphate (dCTP) production in mtDNA synthesis and replication [23]. Both mechanisms help to restore the available pool of mtDNA for oxidative phosphorylation and energy production

Clinicians and scientists at VHIR (Spain) and CUIMC (USA) have collaboratively contributed to TK2d research and development since the first TK2d patients were described in 2001 [14, 15] (Fig. 3). The collaboration represents a critical mass of individuals with multidisciplinary expertise to interpret clinical findings and to propose and test effective treatment modalities for TK2d and other mitochondrial disorders. Within 2 years of the first reported TK2d cases [14, 15], mitochondrial disease researchers at CUIMC characterized genetic and clinical features of TK2d and described its pathogenesis [16, 17]. TK2d is caused by mutations and deletions in the *TK2* gene on chromosome 16q21 in nuclear DNA. More than 30 pathogenic variants in the *TK2* gene have been identified, most of which are missense mutations (70%) [11] and result in loss of function of the TK2 enzyme, which phosphorylates deoxythymidine (dT) and deoxycytidine (dC) to deoxythymidine monophosphate (dTMP) and deoxycytidine monophosphate (dCMP), respectively (Fig. 4). These deoxynucleoside monophosphates are consecutively phosphorylated to the deoxynucleoside triphosphates (dNTPs) necessary for replication of mtDNA [11]. Ensuing mtDNA depletion/multiple deletions lead to inadequate production of mitochondrial respiratory chain complexes needed for mitochondrial oxidative phosphorylation, the main source of cellular energy [18]. Genotype–phenotype correlations were characterized in two mouse models of TK2d. Researchers at CUIMC developed a *TK2* H126N knockin mouse model of TK2d based on observed clinical pathogenic phenotypes [19, 20]. That same year, Swedish researchers independently characterized a *TK2* knockout (*TK2*^−/−^) mouse model [21]. Phenotype in the mouse models recapitulated clinical presentation of severe mtDNA depletion in skeletal muscle, leading to mitochondrial myopathy. Taken together, these data delineated the pathogenesis of TK2d and suggested targeted nucleoside therapy as a plausible treatment modality to restore mitochondrial function by restoring available pools of dNTPs.

Proof-of-concept using nucleotide therapy was first published in mouse models in 2014 [22], paving the way for establishing dCMP/dTMP as the first effective pharmacologic treatment strategy for TK2d in human clinical studies [12]. The preclinical results with dTMP/dCMP in TK2-deficient mice encouraged treatment of the first Spanish patient with dTMP/dCMP, and soon thereafter a second patient was treated in the USA [5, 12]. Further studies demonstrated that, when administered orally, the active compounds are not the monophosphates dTMP/dCMP, but the corresponding nucleosides dT/dC [5, 23]. Pharmacologically, nucleoside therapy restores mitochondrial function through both a cytosolic nucleoside salvage pathway and residual TK2 activity within the mitochondria (see Fig. 4). Approval for the use of nucleosides in humans under compassionate use by the Spanish Drug Agency (AEMPS), driven by CSURs-NMD at H12O and HUVR, led to treatment of approximately 30 patients in Spain between 2014 and 2018 [5, 12]. These clinical data demonstrate the efficacy of nucleoside therapy in slowing or reversing disease progression and prolonging survival among patients with TK2d internationally, with more than 40 individuals currently under treatment in the USA, Central and South America, Eastern Europe, and Asia. Nucleoside therapy was licensed with industry stakeholders in 2016, based on patented intellectual property with Columbia University and the VHIR. PRIME (PRIorityMEdicines) designation was granted by the EMA in 2018 for the product under development, MT1621, which is a combination product of two nucleosides, deoxycytidine (dC) and deoxythymidine (dT), intended as a nucleoside therapy via a calculated dose of combination product (dC/dT) for the treatment of TK2d (Fig. 4). MT1621 was granted Orphan Drug designation by both the US-FDA and the EMA. Breakthrough Therapy Designation was granted by the US-FDA. Clinical studies are under way to obtain regulatory approval for worldwide availability of MT1621 for patients with TK2d.

In parallel with drug development, three seminal papers describing the natural history of TK2d were published in 2018 and 2019 (Fig. 3). These papers represented important milestones in characterizing the natural history of TK2d. Case study and case series reports published over approximately 2 decades were consolidated into the two 2018 publications describing the clinical phenotype and spectrum of disease [8, 10]. A year later, as a consequence of Spanish success in identifying TK2d patients, Domínguez-González et al. published a description of late-onset TK2d based on 18 patients, 16 of whom were identified in Spain. The late-onset form, which accounted for about 20% of cases, was first identified in 2010 as a slower progressing form of disease [11]. Improved genetic testing enabled the characterization of late-onset TK2d in 2013 [24]. These papers led to publication of the first proposed diagnostic and treatment algorithms [7, 8, 10, 11].

### Diagnosis

Detailed characterization of the natural history of TK2d has informed diagnostic algorithms and improved diagnostic accuracy within the CSUR framework in Spain. Typically, patients with TK2d in Spain would initially present to their primary care physician, but general practitioners cannot directly refer patients to CSURs. Patients presenting with muscle weakness are first referred to the general neurology unit or to a neuromuscular neurologist at a local center. If patients do not receive a definitive diagnosis, or if the case shows high complexity, then patients with myopathy are referred to a CSUR-NMD. An appointment is scheduled within 15 days after a simple form is filled out to indicate referral to a CSUR.

TK2d typically manifests as pure muscle weakness, a tissue-specific phenotype that is quite different from most infantile mitochondrial syndromes that exhibit multisystemic involvement and are usually referred to CSURs for inborn metabolism errors. Therefore, in our experience in Spain, patients with TK2d are identified for the first time in neuromuscular units, where a muscle biopsy is frequently performed to guide the diagnosis of patients with muscle symptoms. In patients with TK2d, the biopsy confirms the mitochondrial origin of the disorder, thus directing the subsequent genetic study. If a muscle biopsy is not performed, the use of next-generation sequencing (NGS) approaches using phenotype-driven gene panels (many of which do not include the *TK2* gene) may focus attention on analysis of genes involved in non-mitochondrial myopathies and muscle dystrophies, consequently not reaching the diagnosis. However, as the use of whole exome sequencing (or, in the future, whole genome sequencing) becomes more widespread, diagnosis may be reached via these techniques alone [11, 25]. Once the *TK2* gene has been implicated and a diagnosis of TK2d is in place, a muscle biopsy or assessment in other tissues (e.g., urine epithelial cells, fibroblasts, white blood cells) is not mandatory because the associated metrics (e.g., respiratory chain analysis, mtDNA quantification, TK2 activity from skeletal muscle biopsy specimens) are not essential for management of the disease [7, 26]. Circulating biomarkers such as GDF-15 and/or FGF-21 may also complement molecular and genetic diagnoses, as these metrics provide additional information about monitoring disease progression and pharmacodynamic response to treatment [27].

Occasionally, a patient is diagnosed with mitochondrial myopathy due to muscle biopsy findings, with no deeper exploration into the genetic cause. This could be attributed to the fact that reaching an accurate genetic diagnosis is not easy in the field of mitochondrial disorders (including those manifesting with myopathy) because of involvement of more than 300 genes encoded by nuclear and mitochondrial genomes [28]. Furthermore, this approach has been favored due to lack of treatments for these disorders, but the availability of new disease-modifying therapies has changed the diagnostic and therapeutic landscapes by introducing disease-tailoring therapies that require identification of a genetic defect [29].

Currently, access to genetic studies in the SNS is widely available; hence, we sometimes find patients with mitochondrial disease who are referred from the local neuromuscular unit to the CSUR-NMD unit after already receiving a genetic diagnosis. On the other hand, when a referring physician sends a muscle sample to a laboratory with expertise in mitochondrial diseases, the entire diagnostic process, including thorough genetic testing (i.e., both mtDNA and nuclear encoded gene analysis), is performed without the need for the referring doctor to specify the tests to be performed. This makes it easier for a definitive genetic diagnosis to be reached, even if the doctor sending the sample is not an expert in mitochondrial diseases.

**Fig. 5:**
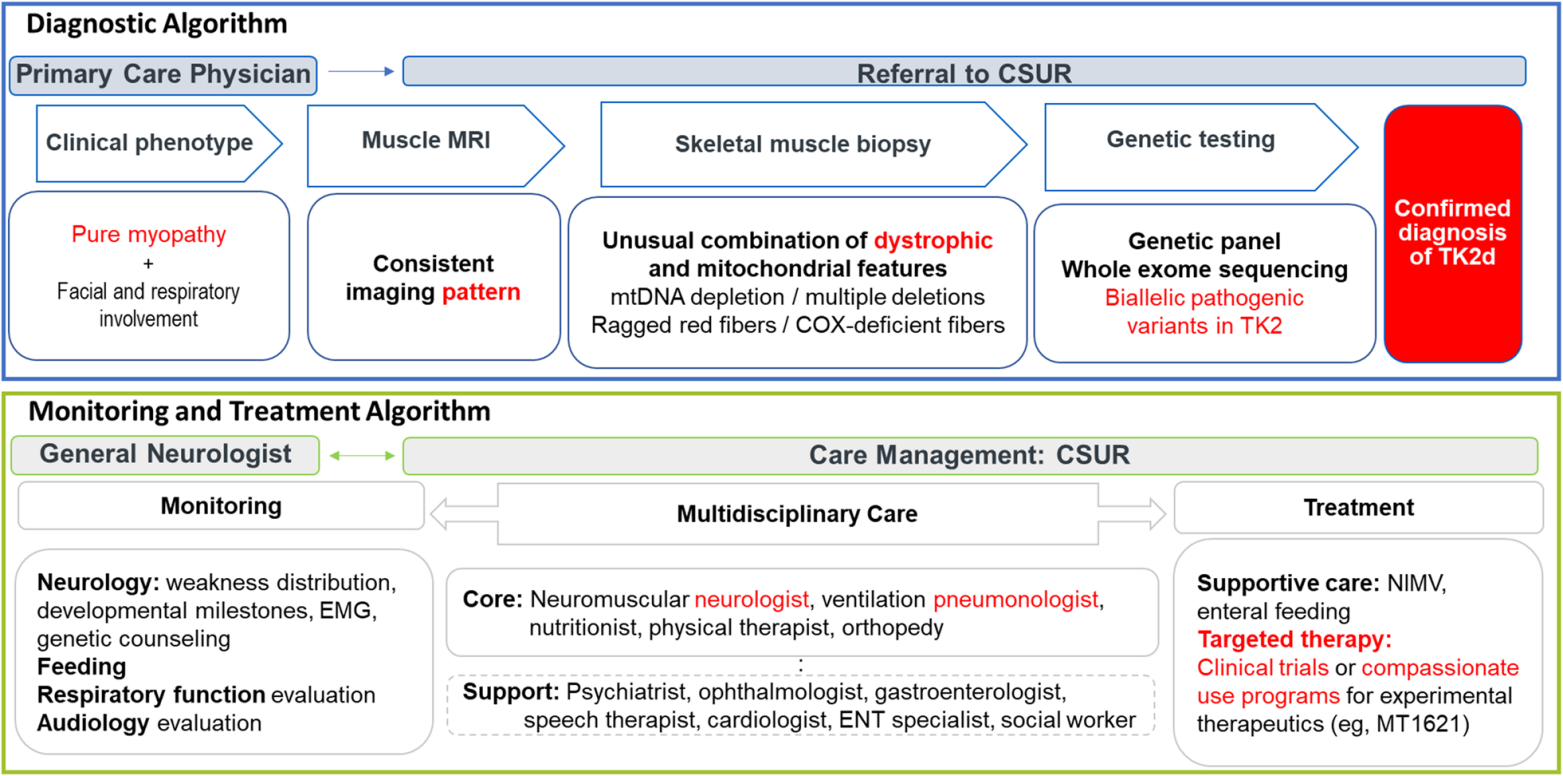
Proposed TK2d diagnostic algorithm [9, 28, 29]. COX, cytochrome C oxidase deficiency; CSUR, National Reference Centers; EMG, electromyography; ENT, ear, nose, and throat; MRI, magnetic resonance imaging; mtDNA, mitochondrial DNA; NIMV, non-invasive mechanical ventilation; TK2d, thymidine kinase 2 deficiency

Clinical guidelines specific to TK2d are under development, but an operational diagnostic algorithm is in place at reference centers in Spain based on consolidation of all published case reports and case series, harmonized with guidelines recommended by the EURO-NMD Muscle Diseases and Mitochondrial Diseases Working Groups (Fig. 5, upper panel) [11, 30–32]. Identification of *TK2* as a causative gene has enabled genetic approaches to molecular diagnosis, including whole exome sequencing, in addition to the mitochondrial diagnostic panel of genes related to mtDNA maintenance after signs of mitochondrial dysfunction are detected on muscle biopsy [4, 15, 30, 32, 33].

### Monitoring and treatment

Patients with TK2d require multidisciplinary care. Reference centers play a crucial role in optimizing access to care [1]. After confirming a diagnosis, CSURs consolidate resources to optimize patient access to care [2, 6] (Fig. 5, lower panel). CSURs consolidate care teams and link patients with appropriate specialists, including physical therapists, speech therapists, pulmonologists, metabolic specialists, and neuromuscular specialists [11, 30, 32]. Consolidating care within the CSUR framework harmonizes patient care, minimizing the number of appointments and time lost from work for the primary caregiver while avoiding contradictory recommendations. CSURs also play a pivotal role in patient education, including genetic counseling for parents, siblings, and prospective parents with late-onset TK2d. CSURs can recommend patient associations for psychosocial and emotional support for patients and their caregivers and families.

### Equity of access

The SNS is fully public, which represents a crucial aspect of care for patients with TK2d and other rare mitochondrial myopathies. Ensuring equity of access to care removes any economic or social barriers that could hinder access of certain patients to the healthcare system and to highly specialized CSURs [6]. The SNS facilitates universal and free access to specialists of any discipline, along with complementary testing and therapeutic procedures for any Spanish citizen and everyone holding the Spanish Health Card. The Spanish population is well accustomed to this universal characteristic of the Spanish Health System, established in the early 1970s. As such, there is little hesitation in obtaining medical consultations on any health problem.

### Limitations

In explaining the high rate of identification of TK2d patients in Spain without additional exploratory studies, it is difficult to rule out completely the possibility of a genetic predisposition or founder effect. However, a founder effect is unlikely, given that patients carry different mutations, both homozygous and compound heterozygous [8, 10]. Further, TK2d does not appear to have an ethnic predisposition, although the p.Lys202del mutation, which is the most frequently reported mutation in late-onset cases so far, has been identified only in Spanish patients and patients of Hispanic ethnic background [7]. Finally, carrier frequency by exome analysis is currently unknown in Spain or elsewhere in the world. Thus, disease frequency cannot be accurately estimated or compared to diagnostic effectiveness in Spain.

## Conclusions

The Spanish collaborative model supports the development of a new therapeutic approach for TK2d, a progressive, ultra-rare disease with no approved therapies. Success in TK2d early identification, diagnosis, and treatment in Spain is attributable to (1) Spain’s fully Public National Healthcare System, which enables equity of access to care, and (2) the designation of CSURs-NMD, which homogenize a highly specialized diagnostic process, including muscle biopsy and molecular study. Universal and free access to genetic studies ensures that a detailed understanding of the genetic causes of mitochondrial disease is more available than in countries with private healthcare systems. The Spanish experience in diagnosis and treatment of TK2d is a model for the diagnosis and development of new treatments for very rare diseases within an existing healthcare system.

## Data Availability

Not applicable.

## Abbreviations

CIBERER: Center for Biomedical Network Research on Rare Diseases
CIBERNED: Center for Biomedical Network Research on Neurodegenerative Disorders
CSUR: Major national reference center
CSURs-NMD: CSURs for neuromuscular disorders
CUIMC: Columbia University Irving Medical Center
dC/dT: Deoxycytidine/deoxythymidine
dCK: Deoxycytidine kinase
dCMP: Deoxycytidine monophosphate
dCTP: Deoxycytidine triphosphate
dNTP: Deoxynucleoside triphosphate
dTMP: Deoxythymidine monophosphate
dTTP: Deoxythymidine triphosphate
ERN: EU reference network
EURO-NMD: Mitochondrial Disease Group within the Neuromuscular Disease ERN
GCP: Good clinical practice
H12O: Hospital U 12 Octubre
HUVR: Hospital U Virgen del Rocío
IBiS: Institute of Biomedicine in Seville
imas12: Research Institute Hospital 12 de Octubre
MDDS: Mitochondrial DNA depletion and deletions syndrome
mtDNA: Mitochondrial DNA
NGS: Next-generation sequencing
PRIME: PRIorityMEdicines
SNS: Spanish National Health System
TK1: Thymidine kinase 1
TK2d: Thymidine kinase 2 deficiency
VHIR: Vall d’Hebron Research Institute
